# Archival Isolates Confirm a Single Topotype of West Nile Virus in Australia

**DOI:** 10.1371/journal.pntd.0005159

**Published:** 2016-12-01

**Authors:** Bixing Huang, Natalie A Prow, Andrew F. van den Hurk, Richard J. N. Allcock, Peter R. Moore, Stephen L. Doggett, David Warrilow

**Affiliations:** 1 Public Health Virology Laboratory, Queensland Health Forensic and Scientific Services, Archerfield, Australia; 2 School of Chemistry and Molecular Biosciences, The University of Queensland, St Lucia, Queensland, Australia; 3 QIMR Berghofer Medical Research Institute, Brisbane, Queensland, Australia; 4 School of Pathology and Laboratory Medicine, University of Western Australia, Nedlands, Australia; 5 Translational Cancer Pathology Laboratory, Pathwest Laboratory Medicine WA, QEII Medical Centre, Nedlands, Australia; 6 Department of Medical Entomology, Pathology West–ICPMR, Westmead Hospital, Westmead, Australia; University of Texas Medical Branch, UNITED STATES

## Abstract

West Nile virus is globally wide-spread and causes significant disease in humans and animals. The evolution of West Nile virus Kunjin subtype in Australia (WNV_KUN_) was investigated using archival samples collected over a period of 50 years. Based on the pattern of fixed amino acid substitutions and time-stamped molecular clock analyses, a single long-term lineage (or topotype) was inferred. This implies that a bottleneck exists such that regional strains eventually die out and are replaced with strains from a single source. This was consistent with current hypotheses regarding the distribution of WNV_KUN_, whereby the virus is enzootic in northern Australia and is disseminated to southern states by water-birds or mosquitoes after flooding associated with above average rainfall. In addition, two previous amino acid changes associated with pathogenicity, an N-Y-S glycosylation motif in the envelope protein and a phenylalanine at amino acid 653 in the RNA polymerase, were both detected in all isolates collected since the 1980s. Changes primarily occurred due to stochastic drift. One fixed substitution each in NS3 and NS5, subtly changed the chemical environment of important functional groups, and may be involved in fine-tuning RNA synthesis. Understanding these evolutionary changes will help us to better understand events such as the emergence of the virulent strain in 2011.

## Author Summary

West Nile virus is endemic in Australia, and is considered benign in relation to strains that circulate globally. In 2011, a more pathogenic variant emerged which caused disease in horses. To understand the evolution of the virus, and as a background to the emergence of the pathogenic strain, we used high throughput sequencing combined with bioinformatics tools to obtain an overview of the evolution of the virus over 50 years. A single lineage regardless of the collection site was apparent. This was also supported by the pattern of changes in sequence between the isolates. The most significant finding was that the single lineage nature of the virus’s evolution infers that regional strains circulate for some years before becoming extinct. The regional strains must then be replaced by continual re-seeding, most likely by waterbirds that disseminate the virus across the continent after above average rainfall. There were changes in the nucleotide sequence that had become established at a population level. These were related to the structure of the viral proteins: in particular the envelope protein, the helicase (NS3) and methyltransferase domain of NS5. There were two changes in catalytic domains which may indicate some fine-tuning of replication.

## Introduction

West Nile virus (Family *Flaviviridae*; Genus *Flavivirus*) is a mosquito-borne virus which can cause disease in humans, horses and birds. In humans, the disease can manifest as a fever with rash and, infrequently, neurological disease including meningitis, encephalitis and acute flaccid paralysis [1]. Introduction of West Nile virus into the Americas in 1999 resulted in tens of thousands of cases of human disease causing over 1,500 deaths, and has devastated bird populations [2]. The virus is now globally distributed, with the exception of Antarctica [3]. There are 7 lineages of which two comprise the majority of circulating strains [4]. In Australia, a strain of West Nile virus called Kunjin virus (WNV_KUN_) is endemic [5]. It has historically been considered more benign than strains circulating globally, but can cause rare cases of non-fatal encephalitis in humans [6]. WNV_KUN_ has been included in Lineage 1b [3], and a single Australian topotype was previously inferred on the basis of oligonucleotide fingerprinting [7].

In 2011, the largest outbreak of equine encephalitis in over 30 years occurred in south-eastern Australia. Over 1,000 horses were infected, with a case fatality rate of 10–15% [8]. Surprisingly, only one case of human disease was reported, despite widespread virus activity [9]. The major aetiological agent of the outbreak, WNV_NSW2011_, was subsequently shown to be neuroinvasive, but was less virulent than the highly pathogenic North American WNV_NY99_ strain in mouse models [10]. Hence, this unprecedented outbreak was an opportunity to explore the drivers causing an apparently benign strain to become virulent in horses. Sequencing revealed that WNV_NSW2011_ carried two known virulence markers that were not present in the prototype WNV_KUN_ strain isolated in 1960 [10]. One was a glycosylation site at position 154 of the envelope. A second was a phenylalanine at amino acid 653 in the RNA polymerase protein encoded by the NS5 gene. Further sequencing revealed that all recent strains isolated, with the exception of the prototype strains, carry both these virulence markers [11], and an additional 4 neuroinvasive strains that had been isolated in the previous two decades were identified. These genotypic changes may be fully or partially responsible for the observed increases in virulence. However, the drivers of the 2011 outbreak still remain unclear and are probably a result of the complex interplay between known, and possibly undetermined, viral genetic virulence determinants; increased fitness in the major WNV_KUN_ vector *Cx*. *annulirostris*; or a convergence of high mosquito and waterbird populations associated with the widespread La Niña driven rain event [11, 12].

Only the original 1960 prototype strains and a handful of coding-complete WNV_KUN_ isolates from the 1980s until the present are currently available in GenBank. As most of the amino acid substitutions of the known determinants of increased neurovirulence of recent WNV_KUN_ strains occurred at least before 1984, there is inadequate sequencing of strains from the early 1960s until that time. In order to better understand the emergence of the neurovirulent strain responsible for the 2011 equine epidemic, and the evolution of the virus, additional sequence data is required. We accessed archival material collected over 5 decades and performed next generation sequencing to generate the data required to discriminate WNV_KUN_ strains at a significantly higher resolution than previous analyses. Changes in the envelope protein were observed, consistent with immune pressure, and also in key replicative proteins which would subtly change the chemical environment of active sites. The analysis revealed a stable genome with a single lineage which was subjected to mostly purifying selection.

## Methods

### Virus collection, isolation and culture

Archival material in the QIMR Berghofer Medical Research Institute collection was obtained over the period from 1960–2011, and was isolated from mosquitoes collected from the Australian states of Queensland, New South Wales, and South Australia (S1 Table). Isolates were stored frozen at -80°C until required. The isolates were passaged twice in C636 *Aedes albopictus* cells prior to sequencing.

### Viral genome sequencing and assembly

Tissue culture supernatants of cultured isolates were processed (excluding the ultracentrifugation step), RNA was extracted, and nucleic acids pre-amplified using sequence-independent amplification (SIA) as described previously [13]. High throughput sequencing was performed on one of two platforms. Library construction and sequencing on the Ion Torrent Personal Genome Machine (PGM™; Thermo Fisher Scientific) was as described previously [13]. The SIA reaction products were subjected to tagmentation, indexing and library amplification following the manufacturer’s recommendations as described in the Nextera XT kit (Illumina). Sequencing was performed on an Illumina HiSeq2500, at the Australian Genome Research Facility. At least 18 million 125 bp paired-end reads (i.e. 9 million pairs) were obtained for each sample. A virus consensus sequence was obtained using Geneious R8 and MRM61C (accession number D00246) as a reference sequence.

### Bioinformatic analysis

Nucleotide sequences were aligned using the MAFFT plugin (algorithm, FFT-NS-ix2; scoring matrix, 200 PAM/k = 2; gap open penalty, 1.53; offset value, 0.123) in the Geneious R8 package [14]. For comparison, an amino acid substitution was defined as a change that was different from at least two separate prior isolates. It was considered fixed when it occurred at least two subsequent times, at least once in a subsequent year and different sampling location. The codon adaptation index (CAI) and expected CAI (eCAI) were calculated as previously described using the server available at http://genomes.urv.es/CAIcal/. For selection analysis, single likelihood ancestor counting (SLAC) and internal fixed effects likelihood (IFEL) analyses were performed as previously described with an optimized substitution model, using the server available at http://www.datamonkey.org/. The data were checked first for evidence of recombination using RDP v4.56 [15]. Unless otherwise indicated, statistical tests used were those incorporated into the software.

For phylogenetic analysis, the optimal substitution model was determined using jModelTest [16] for 88 different models. Using the optimized general time reversible model with invariant sites (GTR+I), a phylogenetic tree was constructed using maximum likelihood in MEGA v7.0.14 [17] with 1,000 bootstrap replicates. The tree was rooted by setting WNV_NY99_ as the out-group. For molecular clock analysis with time-stamped data, the BEAST v1.8.1 package [18] was used with a log-normal relaxed clock. A chain length of 10,000,000 was run, sampling every 1,000. The best supported tree was obtained using the utility TreeAnnotator and a burn-in of 1,000. The optimal tree was graphically modified in TreeGraph2 [19].

### Protein structure analysis

3D biomolecule graphics were done using the viewer in Geneious R8. PDB files containing atomic resolution data (core, 1SFK; envelope, 2I69; NS1, 4OIE; helicase, 2QEQ; MTase, 2OY0; polymerase, 2HCN) were obtained from the Protein Data Bank (http://www.rcsb.org/pdb/home/home.do). WNV_KUN_ data were used, and, where this was unavailable, WNV_NY99_ was used instead. Coloured projections of sequence conservation onto protein structure were conducted using the ConSurf server (http://consurf.tau.ac.il/2016/).

## Results

### Analysis of virulence determinants and fixed amino acid substitutions

Data from 31 isolates were assembled and the consensus sequences aligned with sequence data from other strains available in GenBank. The alignment revealed a remarkable degree of conservation for an RNA virus over the 50 year collection period. Interestingly, the alignment revealed that the substitutions that were previously shown to be associated with virulence [11] had been present for many years. The phenylalanine at 653 of the RNA polymerase, which corresponds to the antagonist of type I interferon-mediated JAK-STAT pathway, was present in all strains with the exception of the MRM61 prototype and its derivatives. The N-Y-S glycosylation motif, present at amino acids 154–156 of the envelope protein, which first appeared in the 1960s, was partially fixed in the sampled population by the 1970s, and completely fixed in all isolates by the 1990s. Therefore, this change probably went to fixation sometime in the 1980s. This conclusion was supported by previous sequencing of the envelope protein of isolates from this decade (J.H. Scherret, unpublished data). Hence, the two known changes from the 1960 prototype strain were mostly likely circulating for at least two decades prior to the 2011 equine outbreak.

There were a large number of amino acid substitutions apparent in the alignment that occurred in single isolates, or a small number of isolates that were collected around the same time, but that failed to go to fixation in the population. These substitutions may be biologically significant for individual strains circulating in a region for a certain period of time. However, the substitutions that go to fixation are most relevant to the understanding of the long-term evolution of the virus. Hence, further analysis will focus on such substitutions.

**Fig 1:**
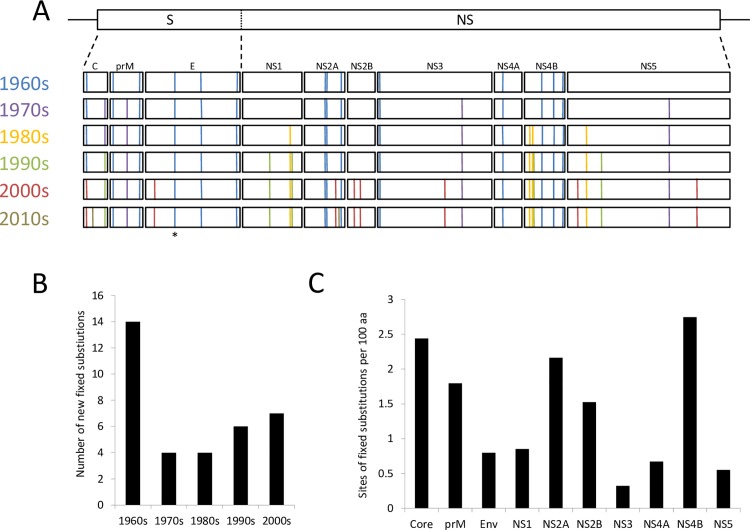
Fixed substitutions in the coding region of WNV_KUN_. (A) The introduction of fixed substitutions in the genome as shown by position. The collection date (indicated by decade using randomly assigned colors) of an isolate where a specific fixed substitution was first detected is also indicated. The substitution resulting in generation of the N-Y-S glycosylation site is indicated (*). (B) The number of fixed substitutions per decade. (C) The fixed substitution density for each processed viral protein.

There were 35 sites where fixed amino acid substitutions occurred in the genome of WNV_KUN_ over the course of 5 decades (Fig 1A; S2 Table). Of note, is the fact that once fixed, these substitutions neither reverted nor were they replaced, and were then permanently maintained in the future virus population regardless of their origin in Australia. For example, the I49V substitution in NS5, previously suggested as an evolutionary marker [11], was identified as one such fixed substitution. This characteristic maintenance of fixed substitutions is indicative of a single long-term lineage of WNV_KUN_ in Australia. The only exception to this observation occurred in two sites of the core protein. Firstly, a S11N substitution that occurred in the 1960s reverted back in the 2000s. Secondly, amino acid 114 of the core protein was substituted twice, once in each of the 1970s and 1990s.

The fixed substitutions were distributed across the genome’s single large open reading frame (ORF) (Fig 1A). There was a minimum of one and a maximum of 6 fixed substitutions per processed viral protein. Changes were not evenly distributed over time (*P* < 0.05; χ^2^ test), with twice as many fixed substitutions occurring in the 1960s than the other decades. This indicated that the period was a hot-spot for non-synonymous nucleotide change. There was some indication of temporal clustering, with all 6 of 7 fixed substitutions in the surface proteins occurring during the 1960s and 70s, and the two fixed substitutions in NS2B occurring during the 2000s (Fig 1B). The fixed amino acid substitution density was lowest for both NS3 and NS5 in comparison with all the other viral proteins (Fig 1C). Both these proteins are critical for RNA synthesis. Therefore, this may indicate some constraint on the replicative machinery which limits the number of possible changes to the protein structure and function before viability is compromised.

### Phylogenetic and molecular clock analyses

**Fig 2:**
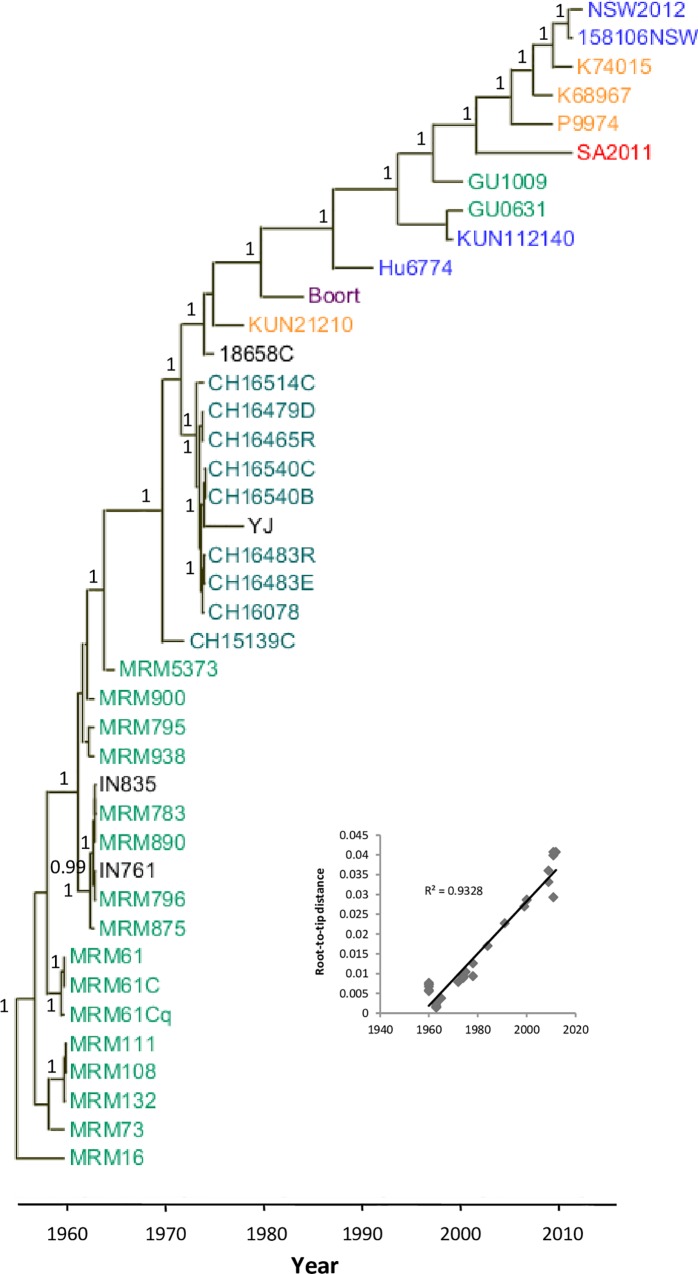
A Bayesian molecular clock analysis based on the alignment of the complete coding region of 31 WNV_KUN_ isolates, and others from GenBank, over a 50 year period. Colours indicate Australian state of origin: northern Queensland, light green; southern Queensland, dark green; New South Wales, blue; South Australia, red; Western Australia, orange; black, unknown origin. Root-to-tip distances plotted against time (inset) showed a strict linear correlation. Additional genome WNV_KUN_ genome sequences included in this analysis were obtained from GenBank: KUNCG (D00246), Boort (KT934796), Hu6774 (KT934797), GU0631 (KT934798), GU1009 (KT934799), SA2011 (KT934803), P9974 (KT934800), K68967 (KT934802), K74015 (KT934801), and NSW2012 (KT934804). Posterior probabilities greater than 0.9 are shown.

A root-to-tip divergence analysis was conducted to determine whether a molecular clock was applicable to the WNV_KUN_ time-stamped sequence data. A strict linear correlation was found between the age of the isolates and the root-to-tip distance (Pearson correlation coefficient r = 0.97, *P* < 0.001; coefficient of correlation R^2^ = 0.93) indicating a molecular clock could be applied (Fig 2). A phylogenetic tree was constructed using BEAST (Fig 2) with an optimized substitution model (GTR+I). This tree morphology was very similar to a tree constructed using the maximum likelihood model with the same data that had not been time-stamped (S1 Fig). Consistent with the conclusions reached from observing the pattern of fixed substitutions, a single lineage was apparent. Multiple clades were present when multiple isolates were collected at a single time point and location. For example, at both Mitchell River Mission in the 1960s and Charleville in the 1970s there were strains that could be grouped in multiple clades. However, after several years, these minor lineages died out to be replaced by the single main lineage. This result, in combination with the analysis of fixed substitutions, strongly suggested a single source or focus from which this lineage is continuously replenished. This may be either a physical and environmentally favourable site, permissive of virus replication; or alternatively, a climatic or seasonal restriction resulting in a bottleneck effect. Finally, from this analysis a rate of 6.924×10^−4^ nucleotide substitutions/site/year was calculated which was comparable to values calculated for West Nile virus [3], and other flaviviruses such as dengue [20], over longer genetic distances.

**Fig 3:**
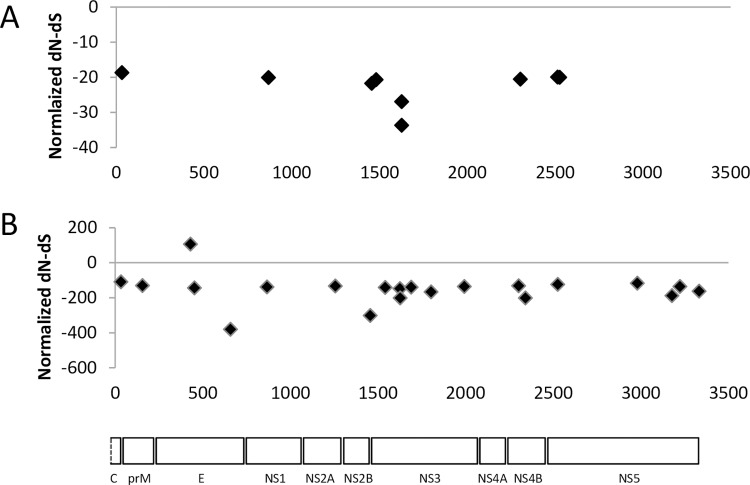
Selection pressure on WNV_KUN_ strains. (A) SLAC and (B) IFEL analysis of WNV_KUN_ polyprotein alignment to detected negative and positive selection pressures measured as normalized *dN*-*dS* across the genome. Statistically significant residues (*P* < 0.05) are shown. Codons 1–3342 (*x*-axis) represent amino acid residues 92–3433 of the polyprotein sequence.

Rates of synonymous (*dS*) to non-synonymous (*dN*) change were calculated across the WNV_KUN_ genome to determine selection pressure. Firstly, the sequences were checked for evidence of recombination using RDP software. The isolate SA2011 was determined to be a possible recombinant (*P* < 0.05 for 6 different models), and was therefore omitted to prevent a problem with the selection analysis. For the other included sequences, the SLAC method detected 9 codons undergoing significant (*P* < 0.05) negative selection (Fig 3A). An alternative method called IFEL, which detects selection along internal branches, detected 20 codons also undergoing negative selection (Fig 3B). Only one codon (codon 231 of envelope) was determined to be under positive selection (*P* < 0.05). However, as this was an asparagine to serine substitution that did not go to fixation, its significance is unclear. Hence, with the exception of possibly one codon in the envelope protein, purifying selection is the major selection pressure being exerted on WNV_KUN_.

### Codon adaptation and the life-cycle of WNV_KUN_

To what extent adaptation to the host’s codon usage influences the life-cycle of WNV_KUN_ is unknown. To explore this question, a measure of codon usage referred as the codon adaptation index (CAI) was used [21]. CAI was calculated for WNV_KUN_ using mosquito (*Culex tritaeniorhynchus*), human (*Homo sapiens*), horse (*Equus caballus*) and bird (*Phalacrocorax carbo*) codon usage data sets (Table 1). The CAI values between the mosquito and all of the vertebrates (human, horse and bird) were significantly different (*P* < 0.001). Hence, there was a codon usage bias towards vertebrate hosts in comparison with the mosquito host.

**Table 1:** CAI values for WNV_KUN_ isolates 1960–2012.

Codon usage set	CAI	eCAI	Kolmogorov-Smirnov test*	CAI/eCAI
*Culex tritaeniorhynchus*	0.592±0.002	0.635	0.023	0.93
*Homo sapiens*	0.759±0.002	0.766	0.032	0.99
*Equus caballus*	0.697±0.002	0.704	0.034	0.99
*Phalacrocorax carbo*	0.691±0.002	0.701	0.032	0.99

* Values less than 0.631 indicate a normal distribution.

To establish whether the observed usage bias was due to codon preference or simply a function of nucleotide composition, an expected value of CAI (eCAI) was determined [22]. This value was calculated using the nucleotide and amino acid frequencies for 500 random sequences at a pre-determined probability (*P* < 0.05), and a ratio of CAI:eCAI greater than one indicated a preference for codon usage. All eCAI values were subjected to a Kolmogorov-Smirnov test and were found to follow a normal distribution. Mosquito CAI/eCAI was not significant (0.93), indicating that usage bias was most likely due to nucleotide composition in that case. CAI:eCAI ratios were for the vertebrates were borderline for significance (0.99) and so it was difficult to be conclusive in that case, but may indicate a codon preference with vertebrates. This result compares with a recent study looking at codon preferences in 449 WNV strains with a clear codon preference towards human usage [23]. The inability to find a clear codon preference in our data may be due to sequence differences between global WNV strains, and WNV_KUN_ strains circulating in Australia.

### Changes in the envelope protein

**Fig 4:**
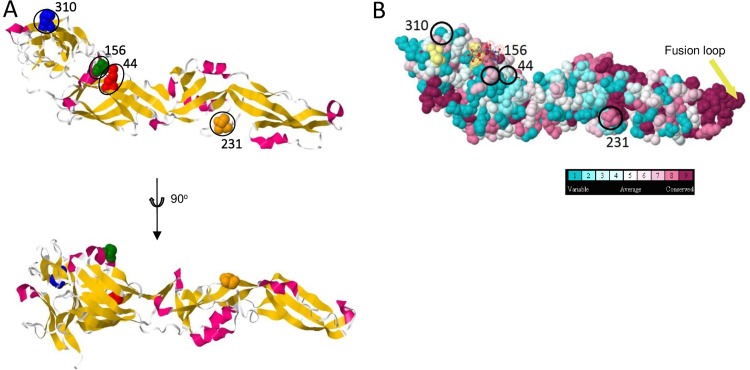
Structure of WNV_KUN_ envelope. (A) Ribbon model (upper) showing fixed substitution residues (space-filled residues: red, amino acid 44; green, amino acid 156; and blue, amino acid 310) and the positively selected amino acid 231 (orange). The structure rotated 90° toward the viewer is also shown (lower). (B) Space filled model with residues coloured according to flavivirus sequence conservation level with corresponding residues indicated.

To gain a better understanding of how fixed amino acid substitutions affected the function of the virus, these changes were related to known WNV protein structures at atomic resolution, where this information was available. For the envelope protein, there were 3 fixed substitutions that could be placed within its structure. The envelope protein is the main viral surface protein and contains the majority of the B cell epitopes of WNV [24]. A K310R substitution in envelope (Fig 4) was within the lateral ridge of domain III (DIII-lr) which contains the major virus neutralization epitope [24, 25]. Another two fixed substitutions that could be placed within the envelope structure (K44R and F156S) were both within domain I, proximal to the interface between domains I and III. The substitution at amino acid 156 is part of the glycosylation motif associated with increased virulence [10]. All of these fixed substitutions were surface accessible, consistent with changes due to selection pressure from humoural immune responses. There was a fourth L483F fixed substitution in the transmembrane domain. However, this is not shown on the figure as it was not included in the structure when this was originally determined. Finally, the positively-selected amino acid at 231 is also shown (Fig 4). It is a relatively conserved residue within the genus *Flavivirus*, suggesting it may have an important biological function.

### Structural and functional changes in the non-structural proteins

**Fig 5:**
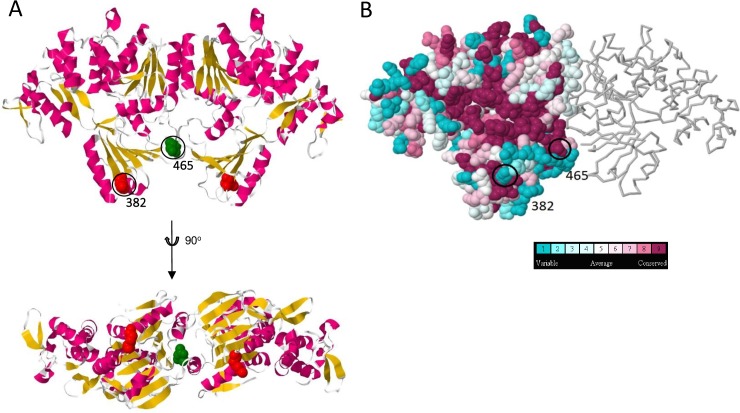
Structure of WNV_KUN_ NS3. (A) Ribbon dimer model (upper) showing fixed substitution residues (space-filled residues: red, amino acid 382; and green, amino acid 465). The structure rotated 90° toward the viewer is also shown (lower). (B) Space filled model with residues coloured according to flavivirus sequence conservation level with corresponding residues indicated.

**Fig 6:**
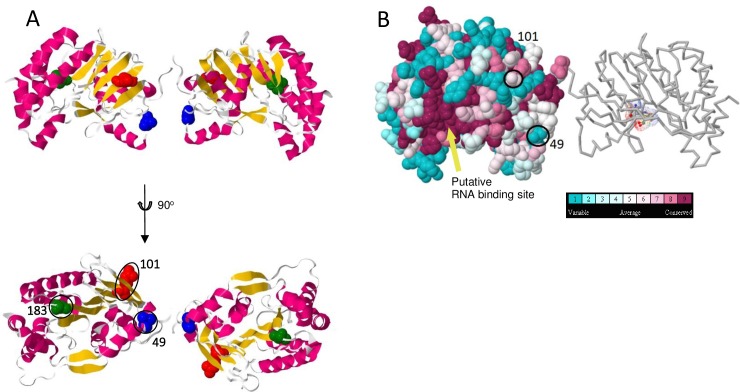
Structure of WNV_KUN_ methyltransferase (NS5). (A) Ribbon dimer model (upper) showing fixed substitution residues (space-filled residues: blue, amino acid 49; red, amino acid 101; and green, amino acid 183). The structure rotated 90° toward the viewer is also shown (lower). (B) Space filled model with residues coloured according to flavivirus sequence conservation level with corresponding residues indicated.

The non-structural proteins are responsible for replication of the virus. To investigate their evolution in WNV_KUN_, the positions of fixed amino acid substitutions were identified in the structures of viral proteins NS1, NS3, and NS5 (S2 and S3 Figs; Figs 5 and 6), as atomic scale information was also available for these proteins. NS3 contains a viral protease and a helicase which facilitates RNA synthesis (Fig 5). There were two substitutions that could be determined within the dimeric structure of NS3: one was K382R and the other was N465S. Amino acid 465 showed a relatively high degree of sequence conservation, suggesting an important functional role. It is immediately adjacent to domain VI of the helicase/NTPase [26]. The substitution was a change from an asparagine to a serine group. Whilst this was a conservative amino acid change, it may have resulted in a subtle shift in the environment surrounding the helicase catalytic groups. The substitution at amino acid 382 was at a variable site, is presumably due to stochastic drift and, therefore, of probably low biological significance.

The NS5 protein is crucial for RNA synthesis, and has two important functional groups: a methyltransferase activity (MTase) responsible for transcript capping (Fig 6), and the RNA polymerase (S2 Fig). A V183I fixed substitution, buried within the protein structure, was immediately adjacent to a residue in the methyltransferase catalytic tetrad (K_61_-D_146_-K_182_-E_216_) [27]. The functional domain of which this residue forms a part is important, as K_182_ directly participates in deprotonation of the 2’-OH group in the ribose. A K182A mutant is attenuated in cell culture further demonstrating the domain’s importance [27]. This valine to isoleucine fixed substitution in WNV_KUN_ at amino acid 183 was another example of a conservative amino acid change, resulting in a subtle change in the chemical environment of the catalytic site. In addition, there were two surface accessible substitutions: one I49V and the other R101K. Both of these were either variable or only slightly conserved, and so were most likely due to stochastic drift. The changes in NS1 and the polymerase domain of NS5 were not near important functional residues, so their structures are provided as supplemental data only.

## Discussion

The most remarkable finding of this work is the stability of the genome over a period greater than 50 years, consistent with other recent observations [28, 29]. Over that period, the picture for West Nile virus in Australia is one of overwhelming purifying selection. There have been a maximum total of 6 fixed substitutions, and in most cases less, for individual viral proteins over that time. Of the changes that have occurred, most are in regions of variable sequence, implying stochastic drift; or where they are in possible functional domains, the changes are conservative in nature, resulting in minor changes at most. From these results, it was hard to deduce any change, or changes, that occurred which have led to enhanced virulence in some strains. Two substitutions were identified previously that were linked to potential enhanced virulence [10]. To better understand the emergence of virulent strains in this study, fixed substitutions were related to protein structure where structural information was available. There will be many other regions left unexplored by this analysis, and therefore, there is much potential to explore this issue further. However, this question is complicated by the possibility that virulence may be related to a single change, or a complex interaction involving changes at multiple sites, making it difficult to reach definitive conclusions.

Our analysis indicates that both of the previously mentioned changes linked to virulence may have been present for over two decades. In particular, the phenylalanine at amino acid 653 of the RNA polymerase was present in all strains except the prototype and its derivatives. A second substitution resulted in the N-Y-S glycosylation motif, and a previous study indicated that most recent WNV_KUN_ strains are indeed glycosylated [11]. As discussed above, this may indicate a trend to glycosylation at this site in recent decades. However, it has been suggested that the absence of the glycosylation site in the prototype and related strains are due to sequence changes occurring during growth in cell culture [9]. Alternatively then, the possibility that the lack of a glycosylation motif in the isolates prior to the 1970s may be a consequence of growth in cell culture cannot be eliminated, without directly sequencing the original material. Unfortunately, due to the length of time since the original collections, this material is no longer available.

RNase-based oligonucleotide fingerprinting over 26 years ago indicated a single topotype for WNV_KUN_ across Australia [7]. The high resolution genome sequencing in this study seems to concur with that early genome analysis. Both the nature of accumulation of fixed substitutions and time-stamped molecular clock analysis strongly suggests a single lineage, and seems to be independent of the site of virus collection. However, due to the sporadic and regional nature of collection, sampling occurred only at one site per year. In order to be conclusive about the observation of a single topotype, multiple samples will have to be obtained at relevant sites around Australia, preferably over multiple years. Complicating this picture, mosquito surveillance suggests circulation of strains different from the main lineage at a low frequency in enzootic regions [11].

Given the probability of a single topotype, it is interesting to speculate on the implications for the virus’s life-cycle and its dissemination. The maintenance of a single long-term lineage requires disseminated strains to eventually die out, to be replaced by another strain which is introduced from a single source. This could be either a location where virus replication occurs in the vertebrate host reservoir or mosquito vector, or alternatively, a local seasonal or climatic restriction which acts as a bottleneck on growth of the virus. In the longer term, these situations would prohibit the circulation of regional strains, and the seeding by a single source. This conclusion from the analysis is in agreement with current hypotheses regarding the epidemiology of WNV_KUN_. The virus is thought to be enzootic in a bird-mosquito transmission cycle in northern Australia, and is disseminated to the southern states after periods of above average rainfall [30]. Hence, the life-cycle presents opportunities for bottlenecks due to both location and climate, and for dissemination.

There is one further implication of a single lineage for WNV_KUN_ in Australia. The 2011 equine outbreak was associated with a more virulent strain of the virus [10], which led to concerns that this strain may establish and also cause serious human disease in the future. If in the long-term WNV_KUN_ strains die out and are re-seeded from a single source, then more virulent local strains may only circulate for several years before being replaced. WNV_KUN_ is enzootic in Western Australia (WA), and most active in the Pilbara region [31]. Isolates collected in WA are less virulent than those associated with the 2011 outbreak [11]. Hence, it is possible that the trend in southern states of Australia may be toward less virulence given sufficient time to replace regional strains.

## Supporting Information

S1 Fig A maximum likelihood phylogenetic tree based on the alignment of the complete coding region of 31 WNV_KUN_ isolates, and others from GenBank, over a 50 year period, and rooted with WNV_NY99_. Colours indicate Australian state of origin: northern Queensland, light green; southern Queensland, dark green; New South Wales, blue; South Australia, red; Western Australia, orange; black, unknown origin. Bootstrap values (10000 replicates) are shown as a percentage.
(TIF)

S2 Fig Structure of WNV_KUN_ NS1. (A) Ribbon dimer model (upper) showing fixed substitution residues (space-filled residues: blue, amino acid 290; and green, amino acid 298). The structure rotated 90° toward the viewer is also shown (lower). (B) Space filled model with residues coloured according to flavivirus sequence conservation level with corresponding residues indicated.
(TIF)

S3 Fig Structure of WNV_KUN_ RNA polymerase (NS5). (A) Ribbon dimer model (upper) showing fixed substitution residues (space-filled residues: blue, amino acid 560; and red, amino acid 719). The structure rotated 90° toward the viewer is also shown (lower). (B) Space filled model with residues coloured according to flavivirus sequence conservation level with corresponding residues indicated.
(TIF)

S1 Table Isolates sequenced in this study. (DOC)

S2 Table Fixed amino acids substitutions. (XLS)

## References

[1] Petersen LR, Brault AC, Nasci RS. West Nile virus: review of the literature. JAMA. 2013;310(3):308–15. PubMed Central PMCID: PMCPMC4563989. 10.1001/jama.2013.8042 23860989 PMC4563989

[2] Lanciotti RS, Roehrig JT, Deubel V, Smith J, Parker M, Steele K, et al Origin of the West Nile virus responsible for an outbreak of encephalitis in the northeastern United States. Science. 1999;286(5448):2333–7. 10600742 10.1126/science.286.5448.2333

[3] May FJ, Davis CT, Tesh RB, Barrett AD. Phylogeography of West Nile virus: from the cradle of evolution in Africa to Eurasia, Australia, and the Americas. J Virol. 2011;85(6):2964–74. PubMed Central PMCID: PMCPMC3067944. 10.1128/JVI.01963-10 21159871 PMC3067944

[4] Mackenzie JS, Williams DT. The zoonotic flaviviruses of southern, south-eastern and eastern Asia, and Australasia: the potential for emergent viruses. Zoonoses Public Health. 2009;56(6–7):338–56. 10.1111/j.1863-2378.2008.01208.x 19486319

[5] Hall RA, Scherret JH, Mackenzie JS. Kunjin virus: an Australian variant of West Nile? Ann N Y Acad Sci. 2001;951:153–60. 11797773

[6] Hall RA, Broom AK, Smith DW, Mackenzie JS. The ecology and epidemiology of Kunjin virus. Curr Top Microbiol Immunol. 2002;267:253–69. 12082993 10.1007/978-3-642-59403-8_13

[7] Flynn LM, Coelen RJ, Mackenzie JS. Kunjin virus isolates of Australia are genetically homogeneous. J Gen Virol. 1989;70 (Pt 10):2819–24. 2552010 10.1099/0022-1317-70-10-2819

[8] Roche SE, Wicks R, Garner MG, East IJ, Paskin R, Moloney BJ, et al Descriptive overview of the 2011 epidemic of arboviral disease in horses in Australia. Aust Vet J. 2013;91(1–2):5–13. Epub 2013/01/30. 10.1111/avj.12018 23356366

[9] Prow NA. The changing epidemiology of Kunjin virus in Australia. Int J Environ Res Public Health. 2013;10(12):6255–72. PubMed Central PMCID: PMCPMC3881112. 10.3390/ijerph10126255 24287851 PMC3881112

[10] Frost MJ, Zhang J, Edmonds JH, Prow NA, Gu X, Davis R, et al Characterization of virulent West Nile virus Kunjin strain, Australia, 2011. Emerg Infect Dis. 2012;18(5):792–800. Epub 2012/04/21. PubMed Central PMCID: PMC3358055. 10.3201/eid1805.111720 22516173 PMC3358055

[11] Prow N, Edmonds J, Williams D, Setoh YX, Bielefeldt-Ohmann H, Suen W, et al Virulence and Evolution of West Nile Virus, Australia, 1960–2012. Emerg Infect Dis. 2016;22(8):1353–62. 10.3201/eid2208.151719 27433830 PMC4982165

[12] van den Hurk AF, Hall-Mendelin S, Webb CE, Tan CS, Frentiu FD, Prow NA, et al Role of enhanced vector transmission of a new West Nile virus strain in an outbreak of equine disease in Australia in 2011. Parasit Vectors. 2014;7:586 PubMed Central PMCID: PMCPMC4280035. 10.1186/s13071-014-0586-3 25499981 PMC4280035

[13] Warrilow D, Watterson D, Hall RA, Davis SS, Weir R, Kurucz N, et al A new species of mesonivirus from the Northern Territory, Australia. PLoS One. 2014;9(3):e91103 Epub 2014/03/29. PubMed Central PMCID: PMC3966781. 10.1371/journal.pone.0091103 24670468 PMC3966781

[14] Kearse M, Moir R, Wilson A, Stones-Havas S, Cheung M, Sturrock S, et al Geneious Basic: An integrated and extendable desktop software platform for the organization and analysis of sequence data. Bioinformatics. 2012;28(12):1647–9. Epub 2012/05/01. PubMed Central PMCID: PMC3371832. 10.1093/bioinformatics/bts199 22543367 PMC3371832

[15] Martin DP, Murrell B, Golden M, Khoosal A, Muhire B. RDP4: Detection and analysis of recombination patterns in virus genomes. Virus Evolution. 2015;1(1). 10.1093/ve/vev003 PMC5014473 27774277

[16] Darriba D, Taboada GL, Doallo R, Posada D. jModelTest 2: more models, new heuristics and parallel computing. Nat Methods. 2012;9(8):772. PubMed Central PMCID: PMCPMC4594756. 10.1038/nmeth.2109 PMC4594756 22847109

[17] Kumar S, Stecher G, Tamura K. MEGA7: Molecular Evolutionary Genetics Analysis Version 7.0 for Bigger Datasets. Mol Biol Evol. 2016. 10.1093/molbev/msw054 PMC8210823 27004904

[18] Drummond AJ, Suchard MA, Xie D, Rambaut A. Bayesian phylogenetics with BEAUti and the BEAST 1.7. Mol Biol Evol. 2012;29(8):1969–73. PubMed Central PMCID: PMCPMC3408070. 10.1093/molbev/mss075 22367748 PMC3408070

[19] Stover BC, Muller KF. TreeGraph 2: combining and visualizing evidence from different phylogenetic analyses. BMC Bioinformatics. 2010;11:7 PubMed Central PMCID: PMCPMC2806359. 10.1186/1471-2105-11-7 20051126 PMC2806359

[20] Pyke AT, Moore PR, Taylor CT, Hall-Mendelin S, Cameron JN, Hewitson GR, et al Highly divergent dengue virus type 1 genotype sets a new distance record. Sci Rep. 2016;6:22356 PubMed Central PMCID: PMCPMC4770315. 10.1038/srep22356 26924208 PMC4770315

[21] Puigbo P, Bravo IG, Garcia-Vallve S. CAIcal: a combined set of tools to assess codon usage adaptation. Biol Direct. 2008;3:38 PubMed Central PMCID: PMCPMC2553769. 10.1186/1745-6150-3-38 18796141 PMC2553769

[22] Puigbo P, Bravo IG, Garcia-Vallve S. E-CAI: a novel server to estimate an expected value of Codon Adaptation Index (eCAI). BMC Bioinformatics. 2008;9:65 PubMed Central PMCID: PMCPMC2246156. 10.1186/1471-2105-9-65 18230160 PMC2246156

[23] Moratorio G, Iriarte A, Moreno P, Musto H, Cristina J. A detailed comparative analysis on the overall codon usage patterns in West Nile virus. Infect Genet Evol. 2013;14:396–400. 10.1016/j.meegid.2013.01.001 23333335

[24] Austin SK, Dowd KA. B cell response and mechanisms of antibody protection to West Nile virus. Viruses. 2014;6(3):1015–36. PubMed Central PMCID: PMCPMC3970136. 10.3390/v6031015 24594676 PMC3970136

[25] Oliphant T, Engle M, Nybakken GE, Doane C, Johnson S, Huang L, et al Development of a humanized monoclonal antibody with therapeutic potential against West Nile virus. Nat Med. 2005;11(5):522–30. PubMed Central PMCID: PMCPMC1458527. 10.1038/nm1240 15852016 PMC1458527

[26] Benarroch D, Selisko B, Locatelli GA, Maga G, Romette JL, Canard B. The RNA helicase, nucleotide 5'-triphosphatase, and RNA 5'-triphosphatase activities of Dengue virus protein NS3 are Mg2+-dependent and require a functional Walker B motif in the helicase catalytic core. Virology. 2004;328(2):208–18. 10.1016/j.virol.2004.07.004 15464841

[27] Zhou Y, Ray D, Zhao Y, Dong H, Ren S, Li Z, et al Structure and function of flavivirus NS5 methyltransferase. J Virol. 2007;81(8):3891–903. PubMed Central PMCID: PMCPMC1866096. 10.1128/JVI.02704-06 17267492 PMC1866096

[28] Ergunay K, Bakonyi T, Nowotny N, Ozkul A. Close relationship between West Nile virus from Turkey and lineage 1 strain from Central African Republic. Emerg Infect Dis. 2015;21(2):352–5. PubMed Central PMCID: PMCPMC4313653. 10.3201/eid2102.141135 25625703 PMC4313653

[29] Shah-Hosseini N, Chinikar S, Ataei B, Fooks AR, Groschup MH. Phylogenetic analysis of West Nile virus genome, Iran. Emerg Infect Dis. 2014;20(8):1419–21. PubMed Central PMCID: PMCPMC4111181. 10.3201/eid2008.131321 25061976 PMC4111181

[30] Marshall ID. Murray Valley and Kunjin encephalitis The Arboviruses: Epidemiology and Ecology. III. Boca Raton, FL: CRC Press; 1988 p. 151–89.

[31] Fitzsimmons GJ, Wright P, Johansen CA, Whelan PI. Arboviral diseases and malaria in Australia, 2007/08: annual report of the National Arbovirus and Malaria Advisory Committee. Commun Dis Intell. 2009;33(2):155–69. Epub 2009/11/03. 10.33321/cdi.2009.33.15 19877534

