# Supplementary figures and images for: Archival Isolates Confirm a Single Topotype of West Nile Virus in Australia
Source: PLoS Negl Trop Dis. 2016 Dec 1;10(12):e0005159. doi: 10.1371/journal.pntd.0005159 (PMC5131910; doi:10.1371/journal.pntd.0005159)

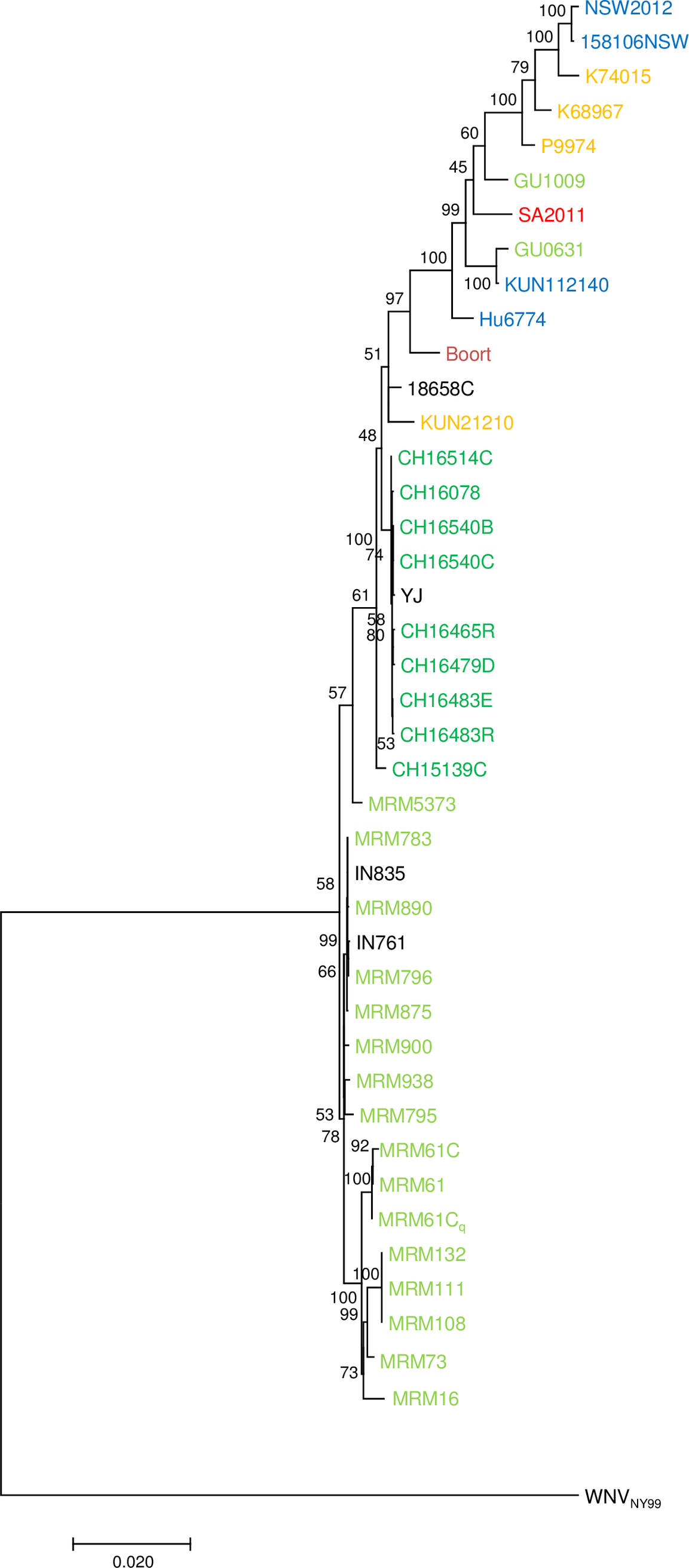

Supplement: S1 Fig — Colours indicate Australian state of origin: northern Queensland, light green; southern Queensland, dark green; New South Wales, blue; South Australia, red; Western Australia, orange; black, unknown origin. Bootstrap values (10000 replicates) are shown as a percentage. (TIF) [file pntd.0005159.s001.tif]

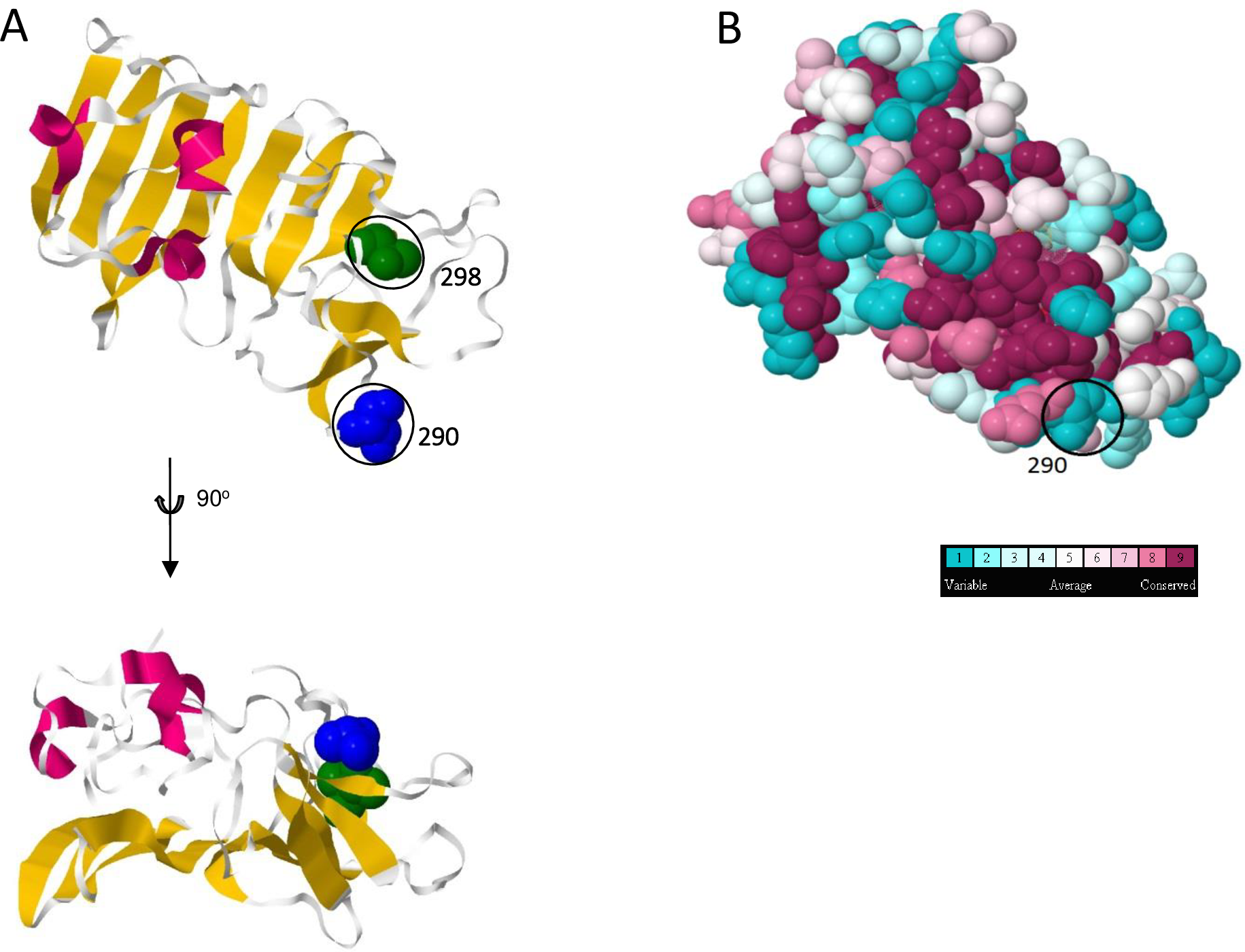

Supplement: S2 Fig — (A) Ribbon dimer model (upper) showing fixed substitution residues (space-filled residues: blue, amino acid 290; and green, amino acid 298). The structure rotated 90° toward the viewer is also shown (lower). (B) Space filled model with residues coloured according to flavivirus sequence conservation level with corresponding residues indicated. (TIF) [file pntd.0005159.s002.tif]

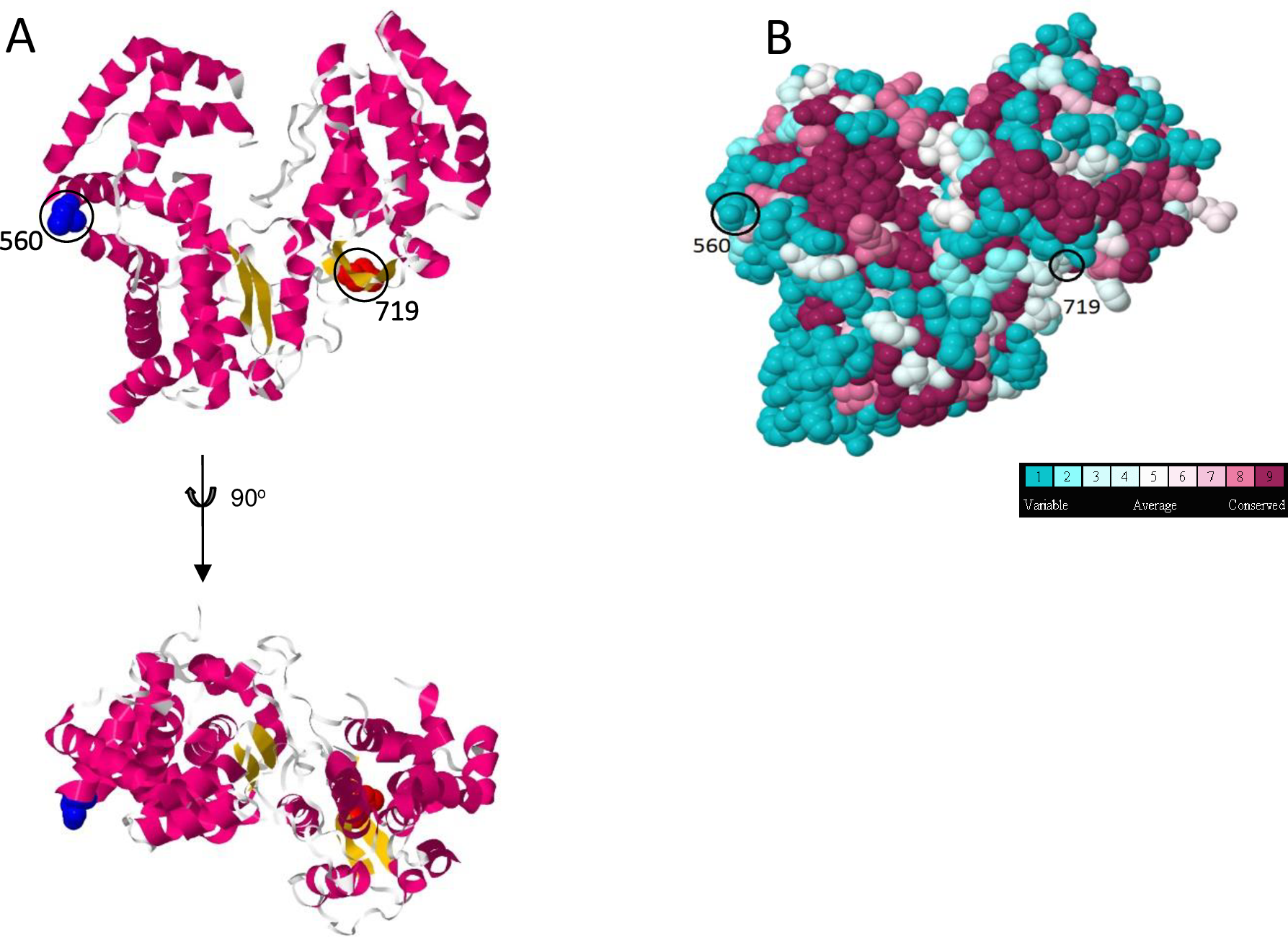

Supplement: S3 Fig — (A) Ribbon dimer model (upper) showing fixed substitution residues (space-filled residues: blue, amino acid 560; and red, amino acid 719). The structure rotated 90° toward the viewer is also shown (lower). (B) Space filled model with residues coloured according to flavivirus sequence conservation level with corresponding residues indicated. (TIF) [file pntd.0005159.s003.tif]
