# Supplementary material for: Archival Isolates Confirm a Single Topotype of West Nile Virus in Australia
Source: PLoS Negl Trop Dis. 2016 Dec 1;10(12):e0005159. doi: 10.1371/journal.pntd.0005159 (PMC5131910; doi:10.1371/journal.pntd.0005159)
Supplement: S1 Table — (DOC) [file pntd.0005159.s004.doc]

S1 Table. Isolates sequenced in this study

| **Isolate** | **Year** | **State collected** | **Species** | **Reference^*^** | **Accession number** |
| --- | --- | --- | --- | --- | --- |
| MRM16 | 1960 | Kowanyama,  Queensland | *Cx. annulirostris* | [1] | KX394396 |
| MRM61 | 1960 | Kowanyama, Queensland | - | - | KX394397 |
| MRM61Cq^†^ | 1960 | Kowanyama, Queensland | *Cx. annulirostris* | QH records | KX394398 |
| MRM73 | 1960 | Kowanyama, Queensland | - | - | KX394399 |
| MRM108 | 1960 | Kowanyama, Queensland | - | - | KX394400 |
| MRM111 | 1960 | Kowanyama, Queensland | - | - | KX394401 |
| MRM132 | 1960 | Kowanyama, Queensland | - | - | KX394402 |
| MRM783 | 1963 | Kowanyama, Queensland | *An. bancroftii* | [2] | KX394403 |
| MRM795 | 1963 | Kowanyama, Queensland | *Cx. squamosus* | [2] | KX394404 |
| MRM796 | 1963 | Kowanyama, Queensland | *Cx. annulirostris* | [2] | KX394405 |
| MRM875 | 1963 | Kowanyama, Queensland | *Cx. squamosus* | [2] | KX394406 |
| MRM890 | 1963 | Kowanyama, Queensland | *An. farauti* | [2] | KX394407 |
| MRM900 | 1963 | Kowanyama, Queensland | *Cx. pullus* | [2] | KX394408 |
| MRM938 | 1963 | Kowanyama, Queensland | *Cx. annulirostris* | [2] | KX394409 |
| MRM5373 | 1965 | Kowanyama, Queensland | *Oriolus flavocinctus*  (bird) | QH records | KX394410 |
| CH15139C | 1972 | Charleville, Queensland | *Cx. annulirostris* | QH records | KX394383 |
| CH16078 | 1974 | Charleville, Queensland | *Cx. annulirostris* | QH records | KX394384 |
| CH16465R | 1974 | Charleville, Queensland | *Cx. annulirostris* | QH records | KX394385 |
| CH16479D | 1974 | Charleville, Queensland | *Cx. annulirostris* | QH records | KX394386 |
| CH16483E | 1974 | Charleville, Queensland | *Cx. annulirostris* | QH records | KX394387 |
| CH16483R | 1974 | Charleville, Queensland | *Cx. annulirostris* | QH records | KX394388 |
| CH16514C | 1974 | Charleville, Queensland | *Cx. annulirostris* | QH records | KX394388 |
| CH16540B | 1974 | Charleville, Queensland | *Cx. annulirostris* | QH records | KX394390 |
| CH16540C | 1974 | Charleville, Queensland | *Cx. annulirostris* | QH records | KX394391 |
| KUN21210 | 1978 | Western Australia | *Cx. annulirostris* | QH records | KX394394 |
| KUN112140 | 1999 | Leeton, New South Wales | *Cx. annulirostris* | QH records | KX394395 |
| 158106NSW | 2011 | Griffith, New South Wales | *Cx. annulirostris* | QH records | KX394382 |
| IN761 | 1963 | Unknown | - | - | KX394392 |
| IN835 | 1963 | Unknown | - | - | KX394393 |
| YJ | 1978 | Unknown | - | - | KX394411 |
| 18658C | 1975 | Unknown | - | - | KX394381 |

^*^QH, Queensland Health; - , no records.

^†^This designation was used to distinguish the isolate from the Berghofer QIMR collection from the previously sequenced MRM61C (accession number D00246).

**References**

1. Doherty RL, Carley JG, Mackerras MJ, Marks EN. Studies of arthropod-borne virus infections in Queensland. III. Isolation and characterization of virus strains from wild-caught mosquitoes in North Queensland. Aust J Exp Biol Med Sci. 1963;41:17-39. Epub 1963/02/01. PubMed PMID: 14028387.

2. Doherty RL, Whitehead RH, Wetters EJ, Gorman BM. Studies of the epidemiology of arthropod-borne virus infections at Mitchell River Mission, Cape York Peninsula, North Queensland. II. Arbovirus infections of mosquitoes, man and domestic fowls, 1963-1966. Trans R Soc Trop Med Hyg. 1968;62(3):430-8. Epub 1968/01/01. PubMed PMID: 4385483.
